# XKR4 Gene Effects on Cerebellar Development Are Not Specific to ADHD

**DOI:** 10.3389/fncel.2017.00396

**Published:** 2017-12-12

**Authors:** Devon Shook, Rachel Brouwer, Patrick de Zeeuw, Bob Oranje, Sarah Durston

**Affiliations:** ^1^NICHE Laboratory, Department of Psychiatry, Brain Center Rudolf Magnus, University Medical Center Utrecht, Utrecht, Netherlands; ^2^Department of Psychiatry, Brain Center Rudolf Magnus, University Medical Center Utrecht, Utrecht, Netherlands

**Keywords:** XKR4, ADHD, cerebellum development, cerebellum, neurodevelopmental disorders

## Abstract

A single-nucleotide polymorphism (SNP) of the XKR4 gene has been linked to Attention-Deficit/Hyperactivity Disorder (ADHD). This gene is preferentially expressed in cerebellum, a brain structure implicated in this disorder. This study investigated the effects of this SNP on cerebellar development in children with and without ADHD. We collected 279 longitudinal T1-weighted structural images and DNA from 58 children with ADHD and 64 typically developing (TD) children matched for age, IQ, and gender. Groups were divided by the XKR4 rs2939678 SNP into A-allele carriers versus subjects homozygous for the G-allele. Cerebellar lobular volumes were segmented into 35 regions of interest using MAGeTBrain, an automated multi-atlas segmentation pipeline for anatomical MRI, and statistically analyzed using linear mixed models. We found decreased gray matter (GM) volumes in ADHD compared to TD children in bilateral lobules VIIIA, left VIIIB, right VIIB, and vermis VI. Furthermore, we found a linear age by gene interaction in left lobule VIIB where subjects homozygous for the G-allele showed a decrease in volume over time compared to A-allele carriers. We further found quadratic age × gene and age × diagnosis interactions in left lobule IV. Subjects homozygous for the G-allele (the genotype overtransmitted in ADHD) showed more suppressed, almost flat quadratic growth curves compared to A-allele carriers, similar to individuals with ADHD compared to controls. However, there was no interaction between genotype and diagnosis, suggesting that any effects of this SNP on cerebellar development are not specific to the disorder.

## Introduction

Neuroimaging studies have implicated the cerebellum in Attention-Deficit/Hyperactivity Disorder (ADHD). However, findings have been inconsistent (Valera et al., 2007) and few studies have examined genetic risk-factors related to these changes. Two studies have suggested that there may be an association between a single-nucleotide polymorphism (SNP) in the XKR4-gene, which is expressed in the cerebellum, and ADHD (Neale et al., 2008; Lantieri et al., 2010). XKR4 codes for a XK-related protein in the XK-Kell blood group complex. While the function of this gene is not well characterized in the brain, XK is highly expressed in the Purkinje cells of the cerebellum in mouse and human tissue (Clapéron et al., 2007; Lee et al., 2007). It has been associated with addiction and substance abuse (Uhl et al., 2008), as well as cognitive deficits, including poor self-restraint, memory, executive function and neuropsychiatric symptoms in McLeod syndrome, a genetically transmitted disorder of the XK-Kell blood group complex (Danek et al., 2001; Jung et al., 2001; Danek and Walker, 2005; Hewer et al., 2007). Additionally, mutations upstream from the XKR4 gene have been shown to mediate response to antipsychotic medication (Lavedan et al., 2009; Fijal et al., 2012). An initial investigation from our lab of XKR4 genotype effects in ADHD showed an interaction with birth weight on decreased total cerebellar volume (De Zeeuw et al., 2012).

Although not all studies of cerebellum volume in ADHD have been consistent, several cerebellar regions have been implicated multiple times in the disorder. Early studies reported smaller total cerebellar volume in children and adolescents with ADHD compared to their typically developing (TD) peers (Castellanos et al., 1996), as well as smaller right cerebellum volume (Durston et al., 2004), and posterior vermis VIII-X volumes (Berquin et al., 1998; Mostofsky et al., 1998; Castellanos et al., 2001). More recent studies have replicated findings in posterior vermis (McAlonan et al., 2007; Yang et al., 2008) and suggested that there may be correlations with symptom severity (Bledsoe et al., 2011; Ivanov et al., 2014), as well as a normalizing effect of medication use (Bledsoe et al., 2009). In addition, reduced volume has been reported for left lobules IV-VI, VIII, IX, X and right lobules IV, VIII, IX (Seidman et al., 2011) and bilateral Crus I (Carmona et al., 2005; Montes et al., 2011). In adults with ADHD, a study using a whole brain ROI approach reported decreased gray matter (GM) in bilateral posterior cerebellum (Makris et al., 2015). Longitudinal studies have suggested that reductions in cerebellar volume in ADHD are stable over development (Castellanos et al., 2002; Mackie et al., 2007; Nakao et al., 2011).

In this study, we set out to investigate whether our previous findings of XKR4 gene effects on the cerebellum were regionally specific, stable over development and, particularly, related to ADHD diagnosis. We assessed the XKR4 rs2939678 SNP and measured regional cerebellar volumes in a longitudinal sample and ran mixed model regression analyses to investigate the effect of the SNP associated with ADHD on cerebellar development. We hypothesized that the associated polymorphism of XKR4 and diagnosis would both be associated with reduced volume, specifically in previously implicated areas, that changes would be stable over development and that there would be an interaction between genotype and diagnosis, where gene effects were greatest for those individuals affected by the disorder.

## Materials and Methods

The Medical Ethical Review Board of the University Medical Center Utrecht approved the study and its procedures.

### Participants

One hundred and twenty-two children (64 TD and 58 children with ADHD) participated in this study from a larger ongoing longitudinal cohort for whom DAT1 and DRD4 had previously been determined (Durston et al., 2005). Groups were matched for age, gender, and IQ (**Table 1**). Written consent was obtained from the parents with full knowledge of the purpose and procedure of the study and children provided written or verbal assent. To confirm inclusion criteria, the DISC-IV parent version (Shaffer et al., 2000) was administered to confirm clinical diagnosis of ADHD or rule out psychiatric morbidity in control participants. In controls, exclusion criteria included history of psychiatric illness in a first-degree relative or any major physical or neurological disorder. In both groups, additional exclusion criteria included any major physical or neurological disorder including dyslexia. Additionally, parents completed the Child Behavior Checklist (CBCL; Verhulst et al., 1996) for a dimensional measure of behavioral symptoms. A four subset Dutch short version of the WISC-R or WISC-III (Wechsler, 2005) was used to assess IQ. At baseline scan, information on medication use was available for 46 of the participants with ADHD, of which 38 reported a history of taking psychostimulants (methylphenidate) and one taking amphetamines (dexamphetamine; **Table 1**).

**Table 1:** Demographic and clinical characteristics [mean (SD)] at baseline.

*Baseline*	TD-GG	TD-A carrier	ADHD-GG	ADHD-A carrier
Gender (M/F)	42/8	13/1	32/5	20/1
Age (year)	10.1 (2.0)	10.3 (1.5)	10.7 (1.9)	10.4 (2.5)
IQ	105.2 (14.4)	107.9 (17.0)	104.2 (16.5)	102.1 (17.8)
**DISC (N)**				
inattentive			6	5
hyperactive/impulsive			8	3
combined			23	13
ODD			13	9
CBCL attention problems	2.8 (2.3)	2.8 (2.4)	9.1 (2.6)	9.5 (3.5)
Medication users			26	12

*GG, homozygous G-allele; TD, typically developing; ADHD, Attention-Deficit/Hyperactivity Disorder; CBCL, Child Behavior Checklist.*

**FIGURE 1:**
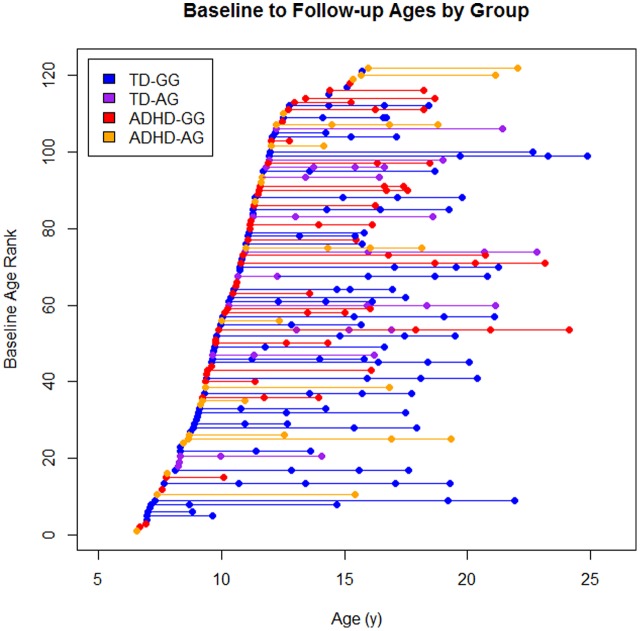
The sample by diagnosis × genotype. Each dot represents a scan and each line represents an individual subject baseline age rank is the ordered age at first scan for each participant.

There were a total of 279 scans available from these 122 children (**Figure 1**). Eighty-one children contributed at least a single follow-up scans (TD: 48, ADHD: 33), 51 had a third scan (TD: 37, ADHD: 14), and 25 had a fourth scan (TD: 21, ADHD: 4).

### Data Acquisition and Processing

A T1-weighted 3D fast field echo scan of the whole head was acquired with 130–150 1.5-mm contiguous coronal slices (Philips Intera; 107 scans) or 160–180 1.2-mm contiguous coronal slices (Philips Achieva; 172 scans) with echo time (TE) 4.6 ms; repetition time (TR) 30 ms; flip angle 30°; field of view (FOV) 256 mm; in-plane voxel size 1 mm × 1 mm.

Scans were processed using MAGeT-Brain^1^, an automated multi-atlas segmentation pipeline for anatomical MRI. Technical details of the segmentation can be found elsewhere (Park et al., 2014). Briefly, MAGeT-Brain uses multiple manually labeled high resolution atlases to generate candidate labels for each voxel. Labels are generated by non-linear registration of randomly selected templates to the anatomical atlases, which are then propagated to the target image and candidate labels are chosen via majority vote. For the current project, the cerebellum was segmented into 35 regions of interest (ROIs) including bilateral and vermis III-X (including VIIB and VIIIA/B) as well as bilateral crus I/II and vermis I, VIIA, and corpus medullare.

### DNA Collection and Genotyping

DNA was collected using buccal swabs as described previously (Durston et al., 2005; De Zeeuw et al., 2012). We selected a SNP in the XKR4-gene that showed nominal significance in two independent association studies (Neale et al., 2008; Lantieri et al., 2010): rs2939678. It was genotyped using Applied Biosystems’ TaqMan SNP assays on ABI Prism 7900 HT real-time thermocyclers. Call rate was >95%, and the SNP did not deviate strongly from Hardy–Weinberg (HW) equilibrium in controls. The XKR4 SNP was recoded to homozygous GG-allele and A-allele carriers (AA and GA) as the number of subjects with homozygous AA-allele carriers was low (one TD, three ADHD subjects). Final groups demographics for baseline are shown in **Table 1**.

### Statistical Analysis

To investigate the relationship between age and volume we used a linear mixed model procedure accounting for uneven interscan intervals, missing data and within subject dependence (Fox, 2002). Analyses were performed using the lme4 package in R (Bates et al., 2015). Each dependent measure of the ith family, jth individual, and kth time-point was modeled as described by Raznahan et al. (2011). Several models including cubic, quadratic and linear age, as well as genotype and diagnosis terms were fit. Intercept, age, diagnosis, and genotype effects were fixed while within person dependence was modeled as a random effect.

The best fit model for each lobule was determined in two steps. First, cubic, quadratic and linear age effects were fit for each lobule using a stepwise approach, where we stepped down to the quadratic model if the cubic model was not significant at *p* < 0.05, etc. Second, the result was fit to three models: (1) Full interaction between diagnosis, genotype and age, (2) a simpler model with two-way interactions terms and, (3) the simplest model including only main effects. The three models were then compared using the Akaike Information Criterion (AIC) to find the model fit that explained the most variance while reducing the number of parameters. In each model, gender and slice thickness were entered as covariates. The interrelated nature of the measures means that traditional methods for correcting for multiple comparisons, such as a Bonferroni correction, are overly stringent for this type of study and there is debate in the literature on how to deal with this issue. In this paper, we chose to not apply any correction, given that our main hypothesis was not confirmed even without correction for multiple comparisons. Lastly, we tested for differences in demographic indices using χ^2^ and 4-group ANOVA where appropriate.

## Results

At baseline, groups were matched for age (*p* = 0.55), IQ (*p* = 0.76), gender (χ^2^ = 2.13, *p* = 0.54) and slice-thickness (χ^2^ = 1.53, *p* = 0.68).

**FIGURE 2:**
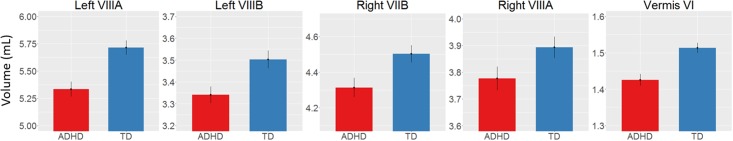
Decreased volume in children with ADHD compared to typically developing children. Y-scale in mL.

**FIGURE 3:**
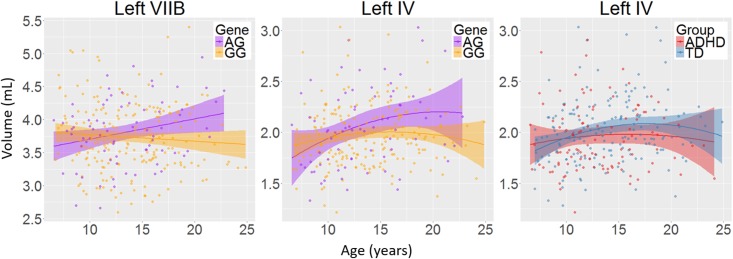
The interactions between age and genotype for left lobule VIIB and IV volumes and between age and diagnosis for left lobule IV volume. Age in years; volume in mL; shade represents 95% confidence intervals.

There was a main effect of diagnosis in five areas (**Figure 2** and **Table 2**), where children with ADHD showed decreased volume compared to TD controls: in bilateral lobules VIIIA (left: *p* = 0.002; right: *p* = 0.026), left VIIIB (*p* = 0.013), right VIIB (*p* = 0.018), and vermis VI (0.024). There were no main effects of genotype. We did find an interaction between genotype and age in left lobule VIIB (*p* = 0.003; **Table 2** and **Figure 3**) and one between genotype and quadratic growth in left lobule IV (*p* = 0.044; **Table 2** and **Figure 3**). In this last area, there was also an interaction between diagnostic group and quadratic growth (*p* = 0.048; **Table 2** and **Figure 3**). There were no interactions between genotype and diagnosis. Nor were there any three-way interactions.

**Table 2:** Best fit regression model for each volume in mL.

Lobule	Model	Intercept	Diagnosis	Gene	D×G	D×A	G×A	D×A^2^	G×A^2^
Left VIIIA	Linear	4.823(0.476)	0.42(0.133)**	-0.101(0.147)					
Vermis VI	Linear	1.475(0.08)	0.073(0.032)*	-0.002(0.035)					
Right VIIB	Linear	3.521(0.377)	0.236(0.099)*	-0.03(0.109)					
Right VIIIA	Linear	3.305(0.304)	0.188(0.083)*	-0.002(0.092)					
Left VIIB	Linear	3.27(0.368)	0.216(0.16)	-0.056(0.131)	-0.049(0.189)	0(0.012)	-0.039(0.013)**		
Left VIIIB	Quadratic	1.97(0.277)	0.198(0.078)*	0.078(0.087)					
Left IV	Quadratic	2.503(0.234)	0.104(0.113)	-0.145(0.094)	0.012(0.128)	0.009(0.009)	-0.034(0.009)	-0.003(0.002)*	0.004(0.002)*

*Parameters for developmental trajectories β (SE). SE, standard error; ^∗^p < 0.05, ^∗∗^p < 0.01; D×G, diagnosis by genotype interaction; D×A, diagnosis by age interaction; G×A, genotype by age interaction.*

## Discussion

The current study set out to investigate whether XKR4 gene effects on cerebellar GM structure were regionally specific, were stable over development and were related to ADHD. Our primary hypothesis was that the XKR4 polymorphism previously associated with ADHD would interact with diagnosis to for structural differences found in ADHD. This was not confirmed. However, we did find decreases in regional cerebellar GM associated with ADHD, as well as protracted developmental trajectories for children homozygous for the G-allele in left lobules VIIB and IV. This latter finding was similar to the current finding in children with ADHD. Overall, our findings suggest that any effects of XKR4 genotype on cerebellar structure are not specific to ADHD, and do not mirror changes associated with the disorder.

We found decreases in regional GM volume in ADHD, including in bilateral lobule VIII, right VIIB, and vermis VI. Lobule VIII is most notably associated with motor and somatosensory processing (Bushara et al., 2001; Grodd et al., 2001; Stoodley and Schmahmann, 2009) and the current result may relate to abnormal motor activity seen in ADHD (Hove et al., 2015). Lobule VII has been associated with language processing, working memory and executive function (Stoodley and Schmahmann, 2009; Buckner et al., 2011) and changes in this lobule may therefore be related to the difficulties in these areas that some individuals with ADHD experience (Doyle et al., 2005; Jonsdottir et al., 2006). One earlier study also reported decreases in volume in vermis VI in ADHD and many studies have reported decreased volumes in posterior inferior vermis VIII-X (Berquin et al., 1998; Mostofsky et al., 1998; Castellanos et al., 2001; Bussing et al., 2002; Goetz et al., 2014). However, these studies have In addition to regional changes in GM volumes in ADHD, we found a genotype by age interaction in left lobule VIIB with subjects homozygous for the G-allele showed a downward growth slope over development and A-allele carriers showed an typically only included children, had small sample sizes or only examined relatively large unsegmented sections of the vermis. upward slope into early adulthood. Furthermore, we found an interaction of age with both diagnostic group and genotype in left lobule IV. Here, the ADHD group showed a more suppressed, almost flat quadratic growth curve compared to TD controls with a peak in late adolescence. Subjects homozygous for the G-allele (the genotype relatively overtransmitted in ADHD) showed a similar trajectory compared to A-carriers. Lobule IV has been associated with motor activity (Stoodley et al., 2012) and anatomical cerebral-cerebellar motor connections (Schmahmann and Pandya, 1997). Differences in this region may be related for the hyperactive motor behavior in ADHD (Pitcher et al., 2003) and motor tics in XKR4 related disorders (Danek et al., 2001).

There are some limitations to our current study. We note that the sample size of the A-allele carrier groups at baseline was relatively small (*N* = 35: TD = 14, ADHD = 21). Furthermore, the sample size at follow-up was also limited due to attrition. This may have led to null findings for some of the higher order interactions. We also note that multiple comparison correction that was not applied in the current study and therefore suggest a need for future replication. Lastly, we suggest future studies examine whether XKR4 expression may influence ADHD development in the cortex.

In summary, we found decreased GM volume in several posterior lobules of the cerebellum associated with ADHD, similar to earlier studies. We found no evidence of main effects of XKR4 genotype on cerebellar volumes, and only limited effects on regional developmental trajectories. Furthermore, there were no interactions between genotype and diagnosis. In all, these findings suggest that any changes in cerebellum associated with ADHD are not due to effects of XKR4 genotype on cerebellar structure.

## Author Contributions

All authors of this manuscript contributed substantially to the scientific process and the writing of the paper. Their contribution included the conception and design of the project, the running of the study, as well as the analysis and interpretation of data. In addition, all authors substantially contributed to either drafting or critically revising the manuscript for important intellectual content and state that they are entirely responsible for the scientific content of this paper.

## Conflict of Interest Statement

The authors declare that the research was conducted in the absence of any commercial or financial relationships that could be construed as a potential conflict of interest.

## Footnotes

1 https://github.com/CobraLab/MAGeTbrain
